# Meeting Future Demand for Chiropractic Services in Hong Kong: A Strategic Manpower Planning Approach

**DOI:** 10.7759/cureus.37481

**Published:** 2023-04-12

**Authors:** Lucina Ng, Valerie Chu, Kary K Lam, Vincent Chan, Rick Lau, Albert Leung, Kingsley Leung, Jacky C Yeung, Christina Cunliffe, Eric Chun-Pu Chu

**Affiliations:** 1 Executive Board, Chiropractic Doctors Association of Hong Kong, Hong Kong, HKG; 2 McTimoney College of Chiropractic, College of Health, Abingdon, GBR

**Keywords:** public education, public health, manpower, chiropractor, chiropractic

## Abstract

Chiropractic treatment in Hong Kong has demonstrated high effectiveness in cases where traditional therapies have failed, with minimal associated adverse events. The growing aging population, prevalence of disabilities, and musculoskeletal conditions have increased the demand for rehabilitation services. Over the past few years, the chiropractic profession has raised awareness of treatment benefits. Providing high-quality training and education, licensing/regulation, interprofessional collaboration, increased accessibility, and research are factors influencing the chiropractic workforce and meeting the population’s health needs. To achieve the number of chiropractors required by Hong Kong for adequate service according to international standards, future efforts could include increased licensing/registration efficiency, expanded coverage of public/private insurance, system integration/interprofessional collaboration, public education, and local research to build evidence and to support workforce growth and acceptance.

## Introduction and background

As the world's population ages and the prevalence of chronic illnesses increases, there is an increasing need for health services that can reduce constraints on activity and involvement. However, there is a global scarcity of medical professionals, particularly those providing rehabilitation services. The World Health Organization (WHO) believes that there is currently a shortage of 7.2 million health workers worldwide, with a health workforce crisis in 83 countries [1]. Coordinated actions are urgently required to alleviate staffing shortages and meet rehabilitation needs. The Global Strategy on Human Resources for Health: Workforce 2030, developed by the WHO, aims to ensure that everyone has access to a sustainable health workforce with the necessary skills and distribution [2]. The accompanying National Health Workforce Accounts provide a framework for monitoring crucial workforce components, including workforce supply, labor market dynamics, and workforce meeting population health needs [2]. Policy and educational measures are required to improve the accessibility and capacity of the rehabilitation workforce, particularly in marginalized areas. Chiropractic care can help reduce activity constraints and participation restrictions caused by musculoskeletal and other chronic health disorders, and is part of the solution to the expanding global need for rehabilitation services [3]. To satisfy this unmet need, deliberate efforts are required to maximize the potential of the chiropractic workforce.

In Hong Kong, chiropractic therapy has been found to be highly effective even after patients have already received traditional therapies [4], and is associated with minimal side effects [5]. According to a survey conducted by the Hong Kong Special Administrative Region’s Census and Statistics Department, 71.7% of chiropractic patients indicated that chiropractic treatment was effective or very effective, and 20% of patients considered it more effective than other treatments [4]. Additionally, a comprehensive study of adverse events in Hong Kong analyzed data from unique patients and 960,140 spinal manipulations. The study found that the incidence of severe adverse events was 0.21 per 100,000 treatments, which indicates that such complications are rare and minimally concerning [5].

Despite the increasing recognition of chiropractic's modern scientific foundation and its integration into healthcare systems worldwide, the chiropractic workforce in Hong Kong still faces obstacles despite several opportunities for growth. This study aimed to explore the current status of the chiropractic workforce in Hong Kong, including key variables, such as the number and distribution of chiropractors. It also examines significant aspects that could influence the growth and development of the chiropractic workforce in the future, including insurance coverage, interprofessional collaboration, public education, and legislative support.

## Review

The chiropractic profession in Hong Kong

History

Chiropractic has a long history in Hong Kong, dating back to before World War 2 [6]. Chiropractors in Hong Kong were formally organized in 1967, and the Chinese terms “spine and nerve doctor" was adopted in 1981 to refer to chiropractors [7]. The statutory regulation of chiropractors began in 1993 with the enactment of the Chiropractors Registration Ordinance [6]. This Ordinance set up the Chiropractors Council of Hong Kong and Hong Kong became the first territory in Asia to officially establish chiropractic as a registered healthcare profession [6]. Only chiropractors listed with the government can legally treat patients and use the title "chiropractor". The chiropractic council is responsible for maintaining professional standards, registering chiropractors, and managing disciplinary matters [6]. The enactment of the ordinance was an important milestone that affirmed the value of chiropractic care as a conservative approach to health and its integration into Hong Kong's healthcare system [7].

Since its inception in 2000, the Chiropractic Doctors' Association of Hong Kong (CDAHK) has been essential for the increased adoption and utilization of chiropractic services in Hong Kong [8]. As the preeminent chiropractic organization representing and advocating for practitioners in the region, the CDAHK has pioneered initiatives to raise public awareness and understanding of the value of chiropractic care for conservative management [8]. Through leadership in professional education, interdisciplinary collaboration, and health policy reform, the CDAHK has effectuated the steady maturation and integration of chiropractic practices in Hong Kong over the past two decades, enabling better delivery of evidence-based, people-centered, interprofessional, and collaborative care [8].

Number of Chiropractors

According to registration data, as of February 28, 2023, there are 315 registered chiropractors in Hong Kong, indicating modest growth over the past decade [9]. However, accurate workforce numbers may be lower because of delays in obtaining data on retired and immigrant chiropractors.

Distribution

Chiropractors in Hong Kong are evenly distributed in Hong Kong Island, Kowloon, and New Territory areas across 185 clinics in 27 mass-transit railway (MTR) stations [8,10].

Scope of Practice

Hong Kong chiropractors are primary care practitioners trained to diagnose and treat neuro-musculoskeletal disorders that benefit from chiropractic care [7]. Chiropractic is a distinct entity of healthcare that focuses on the nervous system and spine, especially the functional and structural changes of the human body [7]. Chiropractors use spinal manipulative therapy and other manual techniques to restore spinal dysfunction and repair soft tissues, with the goal of improving function and reducing pain [7]. In the 2021 Hong Kong Chiropractor Survey, it was seen that 90.41% of chiropractors focused on spinal health, and 87.67% of chiropractors treated neuro-musculoskeletal disorders [11]. Most patients consult chiropractors for musculoskeletal conditions [12].

Chiropractor’s Salary

According to the 2021 Hong Kong Chiropractor Survey, over 50% of chiropractors have a monthly income of 150,000-300,000 HKD per month, and 26.23% reported a monthly income of over $300,000 HKD per month [11]

Influencing factors

Hong Kong’s Growing Aging Population

Hong Kong has one of the fastest-aging populations in the world, and this rate is expected to peak in the next decade. According to the Primary Healthcare Blueprint, from 2021 to 2030, the average annual growth rate of the population aged ≥ 65 years is 4.0%. The number of individuals aged 65 years and above is expected to increase from 1.5 million (20% of the total population) in 2021 to 2.52 million (31%) in 2039. In addition, the percentage of older persons (those aged 80 and older) will increase rapidly from 0.4 million (5%) in 2021 to 0.93 million (11.5%) in 2039 [13]. Aging is also associated with increasing health and social care needs and a higher prevalence of chronic diseases [13]. The percentage of people with chronic health conditions was 31% (approximately 2.2 million) in 2020/21, of which 47% were aged 65 years [13]. Moreover, older people primarily utilize heavily subsidized public healthcare services. This confluence of trends poses major challenges to sustaining Hong Kong's healthcare infrastructure and ensuring access to high-quality treatment for all residents [13]. Efforts to strengthen the geriatric workforce, including expanding chiropractic services for older patients, could help address this pressing socio-medical issue.

Healthcare Needs

The increasing prevalence of disabilities and musculoskeletal conditions, which increase with age, creates a substantial and unmet need for rehabilitation services. Musculoskeletal conditions are the leading cause of disability worldwide [14]. However, the availability of and access to rehabilitation providers who can address activity limitations and participation restrictions are limited in many settings [14]. The Hong Kong Hospital Authority's 2022-23 annual plan estimates that there will be 3.1 million out-patient visits for allied health services and 2.2 million community outreach attendances to support psychiatric and geriatric rehabilitation in the community [15]. The government is pursuing integrated models of specialist outpatient and ambulatory care to reduce reliance on traditional hospital-based services [16]. This program has been expanded to cover several conditions, including musculoskeletal disorders [16]. Chiropractic's non-invasive approach is well suited for managing prevalent musculoskeletal diagnoses. Therefore, as Hong Kong's population shows an increasing occurrence of back/neck pain, osteoarthritis, and other degenerative or post-traumatic conditions that could benefit from chiropractic care, growth in the chiropractic workforce may be warranted to deliver effective treatment and address the escalating prevalence of these conditions.

Awareness

The healthcare ecosystem has been evolving quickly after the coronavirus disease 2019 (COVID-19) pandemic, and the chiropractic profession in Hong Kong has made significant strides in creating awareness about chiropractic treatment and its benefits [17,18]. Through various channels, such as advertisements, social media, online resources, and informational seminars, they have educated people about the effectiveness of chiropractic care [19-21]. Chiropractic organizations in Hong Kong were awarded 31 times between 2014-2022 at the World Spine Day Award, the largest global public health event dedicated to promoting spinal health and well-being [19-21]. The United Nations Children Fund (UNICEF) praises a chiropractor for his commitment to promoting breastfeeding [7]. Additionally, the Hong Kong Chapter of the United Nations' Sustainable Development Solutions Network (SDSN Hong Kong) has recognized Hong Kong’s chiropractic organization, New York Medical Group, for SDG 3, which aims to ensure spinal health and wellness for all [7]. These awards signify a significant increase in awareness of chiropractic treatments in Hong Kong.

Training and Education

Providing high-quality training and education to chiropractors is of utmost importance to ensure that they possess the necessary skills and knowledge to provide effective treatment. Chiropractors from the CDAHK brought the McTimoney College of Chiropractic to Hong Kong, which offers the first chiropractic degree in China, designed to cater to the needs of the Hong Kong community for chiropractic care and to serve the wider population of Hong Kong [22]. This aims to provide chiropractors with the skills and knowledge required to deliver high-quality care. Moreover, about 28.65% of Hong Kong chiropractors hold advanced master’s and doctoral degrees, in addition to their previous degrees, including doctor of philosophy, master of public health, and specialist qualifications in neurology, pediatric, orthopedic, and sports chiropractic [11]. This reflects the emphasis on both initial training and continuing education to ensure that chiropractors are updated with the latest techniques and research in their field. Overall, investing in education and training is crucial for the development of the chiropractic profession in Hong Kong.

Licensing and Regulation

The licensing and regulation of chiropractors are important factors in ensuring that they meet certain standards of practice and that patients receive safe and effective treatments. Hong Kong was the first country in Asia to statutorily register chiropractors [23]. The Chiropractors Council was established under the Chiropractors Registration Ordinance, Chapter 428, in 1993 and served as a registry of chiropractors practicing chiropractic, and the first group of chiropractors was registered in September 2001 [7]. Clear guidelines and standards are well established and enforced to ensure patient safety and maintain the credibility of the profession. However, sick leave certificates issued by chiropractors are still not recognized by the Employees' Compensation Ordinance of Hong Kong, which may result in additional medical expenses for chiropractic patients seeking worker's compensation [23].

Collaboration with Other Healthcare Professionals

Hong Kong chiropractors have been receiving referrals from other healthcare professionals such as medical doctors (18.82%), physiotherapists (9.41%), and acupuncturists (9.02%), who are essential for providing comprehensive care for patients [11]. According to a study on the relationship between chiropractor traits and patient referrals, chiropractors receive an average of 2.5 referrals per week from general practitioners. Chiropractors who specialized in geriatrics and saw more new patients each week reported receiving more referrals [24]. This can also help to build trust and credibility in the chiropractic profession.

Accessibility

Making chiropractic treatment readily available and accessible to patients is important to increasing the demand for the service. According to the Hong Kong Chiropractic Association website, there are 186 chiropractic clinics across Hong Kong Island, Kowloon, and new territories offering flexible appointment times and accepting all payment options [8,10]. Two clinics are also open on Sundays to ensure patient accessibility.

Research

Research on chiropractic effectiveness and outcomes is critical for establishing the credibility and demands of the profession. A search of literature indexes, including Google Scholar, Index to Chiropractic Research, and PubMed, showed that the chiropractic research volume has increased by 2100% from 2015 to 2022 (from two to 42 studies). High-quality studies from Hong Kong, such as the 2023 analysis of adverse events associated with spinal manipulation in Nature Publication, have gained recognition in the healthcare field [5]. Hong Kong author, Chu EC (New York Medical Group), has been tabled as one of the top 10 most prolific authors of chiropractic case reports in a review by Trager and Dusek [25]. When chiropractic research in Hong Kong is published in reputable journals and receives recognition [26], it lends further weight to the evidence base and the value of chiropractic services. Continued research conducted in Hong Kong's chiropractic profession and system could help raise awareness and utilization of chiropractic care to address regional health needs.

Cultural Attitudes Towards Non-Drug Approaches

While cultural attitudes toward manual therapies in Hong Kong present both challenges and opportunities for chiropractic acceptance and use, public education initiatives may help address misconceptions. The Chiropractic Children's Foundation has provided chiropractic education in 348 local schools, training 1000 teachers in all schools and reaching over 400,000 students [17,27]. Such programs teach the benefits of chiropractic care and early detection of spinal problems. Educational workshops for the public also aim to equip participants with knowledge of their roles in health and wellness [17,27]. Ongoing public education regarding chiropractic effectiveness and its potential to address the rising burden of musculoskeletal conditions could increase the understanding and utilization of chiropractic services in Hong Kong.

High Out-of-Pocket Costs for Patients

Owing to the high demand for chiropractic patients and the limited supply of the chiropractor workforce, the out-of-pocket costs of chiropractic care have increased, which may create a significant barrier to access for much of Hong Kong's population. The average fees for chiropractic services have increased by 105% from 2013 to 2021 (450 HKD to 925 HKD) [4,11]. Medical Claims Statistics 2019 from the Hong Kong Federation of Insurers also reported similar data, where the chiropractic fee (812 HKD) was the highest among medical specialists (793 HKD), physiotherapists (534 HKD), and Chinese medical practitioners (429 HKD) [28]. It accounts for 2.3% of total medical claims, whereas the average reimbursement is only 70% [28]. As private insurance plans and public health coverage are limited, access to chiropractic services is also consequently restricted.

However, recent policy developments have suggested an increase in the acceptance of chiropractic. The Elderly Health Care Voucher Scheme subsidizes the use of designated private healthcare providers, including chiropractors, by elderly patients [29]. Although coverage may not meet the full fees, such initiatives could increase access to chiropractic services for those in need. Additional insurance coverage and subsidies would further facilitate primary healthcare clinicians’ access to, and help realize, the potential benefits of chiropractic therapy.

Limited Inclusion of Chiropractic Services in Public Healthcare System

The limited inclusion of chiropractic services in Hong Kong's public healthcare system results in inefficiencies and barriers to workforce growth [30]. Patients with musculoskeletal and neuromusculoskeletal conditions must navigate unnecessary waits and referrals to access chiropractic care, increasing system burden [30]. In addition, as Hong Kong chiropractors often refer patients to nearby hospitals for urgent management [31-47], the current system delays immediate emergency care. The lack of chiropractic exposure in medical education also perpetuates solid system and inter-professional barriers. Integrating chiropractic care into public health coverage and professional education could reduce unnecessary demands on the healthcare system and workforce, enabling a more coordinated model of care with interprofessional collaboration in which different disciplines work to their full scope to deliver optimal outcomes.

Future directions in manpower planning

Increased Registration/Licensing to Establish Accurate Workforce Numbers and Standards

Increasing the efficiency and transparency of chiropractic registration and regulatory boards could help develop accurate manpower flow and maintain high practice standards. The appointment process for the Hong Kong Chiropractic Council could be made more transparent and merit-based to avoid biased appointments including relevant public health experiences, advanced education, and academic contributions. Currently, Chiropractic Council members are represented by a small, affiliated group of chiropractors appointed by the government [6], who may not always take public opinion into account [8]. With a more representative council membership that includes chiropractic organizations and education programs, the public could have greater input and representation in council decisions. Having a responsible council member can streamline the licensing application process, and new chiropractors can start practicing and earning an income sooner after graduation, which could increase their incentives to enter the profession. Efforts to improve registration and licensing procedures could support the growth of a qualified chiropractic workforce to meet the health needs of the population.

Expansion of Chiropractic Coverage in Public and Private Insurance Plans

Policymakers can support chiropractic workforce growth through measures such as expanding the coverage of chiropractic services in public and private health insurance plans [22]. Integrating chiropractors into the healthcare system and increasing access to their conservative approach could offer patients more treatment options and potentially alleviate the burden on orthopedic services. Incentivizing patients with musculoskeletal conditions to access private chiropractic clinics could decrease wait times and demand for the public system [48,49]. Coverage and incentives for chiropractic care could help address the rising burden of musculoskeletal conditions, enabling individuals to access the most appropriate care for their condition and allowing the efficient use of health system resources.

Integration into Public Healthcare System and Interprofessional Collaboration

Integrating chiropractic into Hong Kong's public health system and promoting interprofessional collaboration could increase access to chiropractic services and enable a more coordinated model of care [7,22,49]. Including chiropractors in the public coverage and providing chiropractic services at government clinics and hospitals can provide patients with additional options for conservative musculoskeletal management. Inter-professional education and practice can break down the barriers between chiropractors and medical doctors, enabling them to work together in their full scope of practice for optimal outcomes. Such integration and collaboration could make the most of the available health resources available to address the growing burden of musculoskeletal disease, potentially reducing unnecessary demands on the healthcare system.

Public Education About Chiropractic

Insurance companies, the government, and chiropractors should work together to improve public education regarding chiropractic. The field remains poorly understood in Hong Kong despite its potential to address musculoskeletal conditions. Educational campaigns could teach chiropractors to regulate health professionals who use manual techniques to treat neuromusculoskeletal problems and provide evidence of its effectiveness [7,49]. A better understanding of the benefits of chiropractic care can increase its acceptance and use [7,49]. Such public education efforts, especially if undertaken collaboratively, could help Hong Kong's population access the chiropractic services that they need to manage musculoskeletal health conditions.

Research on Chiropractic Practice and Outcomes in Hong Kong Population to Build Evidence Base

Chiropractors continue researching chiropractic practices and outcomes, particularly in Hong Kong [50-70]. High-quality studies evaluating chiropractic effectiveness, risks, and cost-effectiveness for common musculoskeletal conditions in Hong Kong could provide a local evidence base for their value. Research published in reputable journals lends further credibility to the field in the healthcare profession. Such research could inform policy decisions on including chiropractic in public coverage and interprofessional collaborations. Continued research on chiropractic practices and outcomes could strengthen the foundation for the growth of chiropractic services to meet the health needs of the Hong Kong population.

International, local, and professional academics have not reached an agreement on the estimated manpower project. According to international standards, the ideal chiropractor-to-population ratio is 1:2,588 [71]. Hong Kong's population of over seven million corresponds to approximately 2, 700 chiropractors to adequately meet the region's health needs. However, as manpower projection requires highly data-intensive activity, the utilization of fragmented, incomplete, or unavailable private data may lead to inconsistent results [71]. A 2019 study from the University of Hong Kong concluded that there is an increasing surplus for chiropractors from 2020 by using a utilization-based projection with inpatient and outpatient data that are readily available in the public and private sectors [72]. The CDAHK shared an opposing view of a shortage of chiropractors in manpower planning and the salary report in the 2021 survey [11], where 60% of chiropractors had employment opportunities available before graduation and 31.25% were offered positions within six months after graduation. In addition, the average chiropractor earned 2,700,000 HKD per year [11], compared to 568,816 HKD for physiotherapists [73], and 2,910,202 HKD for orthopedic surgeons [74]. Therefore, we listed the steps that could achieve significant growth in the chiropractic workforce needed to approach the suggested ratio and ensure access to chiropractic care.

## Conclusions

The government should prioritize strengthening and utilizing the chiropractic workforce to meet Hong Kong's healthcare needs while also being consistent with international standards and recognizing the high demand for chiropractors among local professional organizations. Although chiropractic has seen growing recognition in Hong Kong, the profession's size and integration into the healthcare system could expand. This review discusses the key ways in which policymakers and stakeholders can support chiropractic workforce growth, including expanding public coverage of chiropractic services, fostering interprofessional collaboration, increasing public understanding of chiropractic value, and funding research to build a local evidence base. With policy-level efforts to integrate chiropractic into Hong Kong's healthcare system in line with international and professional standards, the chiropractic profession can gain increased acceptance and utilization, contributing to conservative musculoskeletal management and population health.
